# Patient Satisfaction With Minimally Invasive Cardiac Incisions: A Retrospective Single-Centre Survey

**DOI:** 10.7759/cureus.90852

**Published:** 2025-08-24

**Authors:** Oliver M Scarborough, Sparsh Shah, Alexander J Scarborough, Ranjit Deshpande

**Affiliations:** 1 Trauma and Orthopaedics, Manchester NHS Foundation Trust, Manchester, GBR; 2 General Surgery, Glasgow Royal Infirmary, Glasgow, GBR; 3 Plastic and Reconstructive Surgery, Chelsea and Westminster NHS Foundation Trust, London, GBR; 4 Cardiothoracic Surgery, Kings College Hospital, London, GBR

**Keywords:** cardiac surgery, conventional median sternotomy, cosmesis, minimal invasive cardiac surgery, scar satisfaction

## Abstract

Background

Minimally invasive cardiac surgery (MICS) may offer benefits such as reduced intra-operative bleeding, improved postoperative pain and reduction in wound-related complications such as surgical site infection and wound dehiscence, when compared to median sternotomy (MS). Patient satisfaction may be furthered through improved scar cosmesis. However, to the best of our knowledge no study has formally assessed the scar-related satisfaction associated with MICS. This study was designed to analyse the scar-related satisfaction in patients who underwent cardiac surgery via MICS vs MS.

Method

A four-question survey was proposed to our patient cohorts via telephone following verbal consent, at least nine months following their operation. The survey consisted of two open questions regarding postoperative analgesia use and days taken to return to daily activities, one visual analogue scale regarding scar satisfaction (1-10) and one binary question regarding tolerance for scar positioning. Additional patient data including age, gender, duration of surgery, and postoperative length of stay, amongst others, were extracted from our patient database.

Results

Our study shows a reduction in postoperative length of stay (p=0.033) and days taking painkillers (0.049) in the MICS group. Furthermore, scar satisfaction in this group was improved (p=0.032) with a reduction in the number of patients bothered by the location of the scar (p<0.001). 62.5% of the MS group reported that they would have preferred to have had their operation facilitated through MICS.

Conclusion

Little research has been done comparing patient satisfaction in patients undergoing minimally invasive cardiac surgery compared to traditional median sternotomy. Our paper shows that minimally invasive cardiac surgery may confer benefit with regard to cosmesis of scar, hospital stay and overall patient satisfaction. Minimally invasive cardiac surgery can improve cosmesis, as shown by our results, which may help boost body image and confidence.

## Introduction

Minimally invasive techniques have been used in cardiac surgery for over 20 years, since Rao and Kumar [1] first performed aortic valve replacement through a right anterior thoracotomy incision in 1993. Minimally invasive cardiac surgery (MICS) has become more common over the past decade in select patient cohorts [2,3] due to reduced operative trauma, intensive care and hospital stay, and hospital cost with less need for postoperative analgesics compared to traditional midline sternotomy [4,5]. Furthermore, it has been hypothesised that improved outcomes, particularly improved respiratory function, can be achieved in MICS through less spreading of the incision, no interference with the diaphragm, and less tissue dissection [6]. Minimally invasive techniques may have the added bonus of better cosmetic outcomes compared to midline sternotomy incisions, which may be a source of concern for some patients [6].

There have been previous papers that compared various clinical outcomes in traditional sternotomy versus minimally invasive cardiac surgery. However, most of these papers look at operative factors such as bypass time, blood loss and length of hospital stay. Where patients’ satisfaction is assessed, the measured outcomes tend to include pain score, analgesia requirement and time to return to normal work. However, our paper will assess patient satisfaction with the scar itself and convalescence with median sternotomy compared to minimally invasive cardiac surgery.

## Materials and methods

Study design

A total of 80 patients were included in this retrospective single-centre survey. The patients were split evenly with 40 in each group. Initially, 70 patients were identified for inclusion in the MS group. However, 30 patients either didn’t respond to contact or declined to take part. Forty patients were therefore included, and the MICS group was formed to match the same number. In both groups, the earliest operation date was 01/08/2018 and the latest was 29/04/2020. Data was collected retrospectively to allow for a sufficient sample size and allow for both complete healing of the surgical scar and appropriate follow-up. 

Patients were eligible for inclusion in the study if they had any form of cardiac surgery through either a median sternotomy incision or MICS incisions, prior to 1st May 2020 (12 months prior to the date of data collection). All patients were adults aged 16 years or older. Patients were excluded if the intervention served as an emergency procedure or if they had concurrent non-cardiac surgical procedures alongside the primary cardiac procedure. Children less than 16 years of age were also excluded. 

Primary outcomes

To assess patient satisfaction with cosmesis of scars from minimally invasive cardiac surgery, compared with the conventional median sternotomy. 

Secondary outcomes

To analyse any difference in markers of convalescence, including postoperative length of stay, number of days taken to return to daily activities and the number of days for which analgesics were taken postoperatively, and assess if these markers correlate to patient satisfaction. 

Data collection

A four-question survey was developed following a review of the literature and a consensus discussion between consultant cardiothoracic surgeons at our centre (Appendix 1). For patients who underwent median sternotomy, an additional question was included to ascertain if these patients would have preferred a less invasive scar. The survey was then proposed to the two groups of patients who underwent cardiac surgery at a tertiary cardiothoracic centre in London, UK. The patients were contacted by telephone and verbally consented to participate in the survey. All data was anonymised and included no patient identifiable information. The patients were reassured that whether they chose to participate would not influence the care they received going forwards. Additional data surrounding the patient’s demographic and postoperative outcomes were also extracted from the cardiothoracic database. 

Where patients reported they were still taking analgesia for pain relating to the procedure, the number of days was calculated from the date of the operation to the date on which they answered the survey. 

The data was collected for audit and service evaluation purposes only and was not shared beyond the authors of this paper and the statistician. All survey responses and patient data were compiled into Microsoft Excel version 16.49 (Redmond, WA, USA). 

Demographics and details of hospital stay for these two groups of patients were also extracted from the cardiothoracic database by an independent data analyst. Data included patient hospital number, date of birth and age, type of surgery i.e. which valve and any other procedures, duration of surgery (in minutes), length of stay in hospital (in days) and any documented complications.

Statistical analysis

The patient data was statistically analysed using SPSS Statistics version 26.0 (IBM Corp., Armonk, NY, USA). As described by the central limit theorem, the distributions of all data sets were assumed to be normal due to a sample size greater than thirty in each group. Therefore, parametric methods were applied to analysis of all data sets. For all continuous variables, the two-sample unpaired t-test was used to formulate p-values. For binary categorical variables, the Pearson's chi-squared test was used for data sets in which all contingency table values had a count of five or more. Where this requirement wasn’t met for at least one cell in the contingency table, Fisher’s exact test was applied to analyse binary, categorical variables. Univariate analysis was used to analyse the relationship between nominal variables and scaled variables. Multiple regression analysis was used for analysing the relationship between continuous variables and scaled variables. A P-value of less than or equal to 0.05 was considered statistically significant. The applied statistical methods were validated by an independent statistician. All final data was then formulated into tables in Microsoft Excel. 

## Results

Patient demographics, operative details and postoperative length of stay for both groups are shown in Table 1. It shows that the average age of participants was 64 in the MICS group, and 67 in the median sternotomy group (p=0.24). There were more males in the MICS group, however the difference did not reach statistical significance (29 vs 27, p=0.808). More procedures required the application of cardiopulmonary bypass (CPB) in the median sternotomy group (26 vs 37, p=0.05) and the average duration of surgery was lower in the median sternotomy group at 243 minutes vs 297 minutes in the MICS group (p<0.001). The average postoperative length of stay was reduced in the MICS group (8.25 vs 12.3 days, p=0.033). Surgical incisions in the MICS group included mini-thoracotomy, mini-thoracotomy endoscopic, thoracotomy endoscopic, partial sternotomy endoscopic and endoscopic. 

**Table 1:** Demographics and operative details of both groups. All values reported as average ± standard deviation (1 dp) unless otherwise stated. Two-sample unpaired t-test was used to formulate p-values. MICS: minimally invasive cardiac surgery, MS: median sternotomy, CPB: cardiopulmonary bypass

Demographics and operative details	MICS group (n, 40)	MS group (n, 40)	P value
Age (years)	64 ± 10.2	67 ± 12.0	0.24
Gender n, male (%)	29 (72.5%)	27 (67.5%)	0.808
Gender n, female (%)	11 (27.5%)	13 (32.5%)	0.808
CPB used. n, yes (%)	26 (65%)	37 (92.5%	0.05
CPB used. n, no (%)	14 (35%)	3 (7.5%)	0.05
Duration of surgery (minutes)	297 ± 57.5	243 ± 83.4	<0.001
Postoperative length of stay (days)	8.3 ± 4.0	12.3 ± 11.1	0.033

Full survey results are shown in Table 2. It shows that the average number of days taking analgesia was reduced by just under half in the MICS group (9.7 days), with the median sternotomy group reporting 16.4 days (p=0.049). Time taken to return to daily activities was found to be lower in the MICS group with the average being 48.7 days, whereas it took 103.2 days in the median sternotomy group (p=0.023). On average, participants reported a scar satisfaction of 9.2 in the MICS group compared with 8.2 in the median sternotomy group (p=0.032). Furthermore, no patients were unsatisfied about the position of the scar in the MICS group, whereas six patients (15%) in the median sternotomy group were bothered by the location of the scar (p<0.001). Finally, the supplementary question asked to the median sternotomy group alone revealed that 25 (62.5%) of participants would’ve preferred the operation to be facilitated by a smaller incision. 

**Table 2:** Survey results, all values are represented by an average ± standard deviation unless otherwise stated. Two-sample unpaired t-test was used to formulate p-values. MICS: minimally invasive cardiac surgery, MS: median sternotomy

Survey questions	MICS (n, 40)	MS (n, 40)	P value
Time after surgery taking analgesia (days)	9.7 ± 9.4	16.4 ± 19.1	0.049
Time to return to normal daily activities (days)	48.7 ± 55.8	103.2 ± 137.7	0.023
Scar satisfaction level (0-10, very unsatisfied- very satisfied)	9.2 ± 1.2	8.2 ± 2.7	0.032
Does the position of the scar bother you? n, yes (%)	0 (0%)	6 (15%)	<0.001
Does the position of the scar bother you? n, no (%)	40 (100%)	34 (85%)	<0.001
If you could have had your procedure via a smaller incision, would you have wanted to? n, yes (%)	N/A	25 (62.5%)	N/A
If you could have had your procedure via a smaller incision, would you have wanted to? n, no (%)	N/A	15 (37.5%)	N/A

The surgical procedure(s) that the patients underwent in each group are shown in Table 3. In the MICS group, 29 patients (72.5%) had surgery on a single valve only. There were 11 patients (27.5%) who underwent coronary artery bypass grafting (CABG) surgery alone. No patients underwent valvular or CABG alongside another procedure. In the MS group, 19 patients (47.5%) underwent CABG surgery alone. Ten patients (25%) had valvular surgery only, and eight patients (20%) had valvular surgery + surgery on another cardiac site. Four of these patients underwent valve + CABG surgery. 

**Table 3:** Surgical procedures for both groups. CABG: coronary artery bypass grafting, MICS: minimally invasive cardiac surgery, MS: median sternotomy

Surgical procedure	MICS group (n, 40)	MS group (n, 40)
Valve alone	29	10
Valve + aortic root	-	1
Valve + ascending aorta	-	1
Valve + aortic root + ascending aorta	-	2
CABG alone	11	19
CABG + valve	-	4
CABG + ascending aortic arch	-	1
Ascending aortic arch alone	-	2

The documented complications for both groups are shown in Table 4. Interestingly, the number of complications was higher in the MS group, with 21 patients (52.5%) suffering some form of complication. This was compared with just 14 patients (35%) in the MICS group. For both groups, arrhythmia was the most common complication. This occurred in 32.5% of the MS patients and 22.5% of the MICS patients. There were no documented infections in the MICS group and two (5%) in the MS group. 

**Table 4:** Documented complications for each group. These have been broadly split into arrythmia, pulmonary, infection and other. ‘Other’ complications included acute kidney injury, stroke, thromboembolism and delirium. MICS: minimally invasive cardiac surgery, MS: median sternotomy

Complication(s)	MICS group (n, 40)	MS group (n, 40)
Arrythmia only	5	11
Pulmonary only	2	2
Arrythmia + pulmonary	2	2
Arrythmia + other	2	-
Pulmonary + infection	-	2
Pulmonary + other	1	2
Other	2	2
None	26	19

## Discussion

Length of stay

In our study, we found that the postoperative length of stay favoured the MICS group (8.25 days vs 12.3 days, mean difference = 4.05 days). Reduced length of stay in minimally invasive cardiac surgery has been found to be the primary driver of decreased hospital costs. In their multi-centre analysis of 367 patients, Grossi et al. compared hospital costs between patients undergoing mitral valve repair via either an antero-lateral thoracotomy approach (ALT) or median sternotomy. They found the total mean hospital cost to be decreased by $8289 (17.2%) in the ALT group [7]. Alongside the reduced postoperative length of stay, the same study found that the observed reduction in hospital costs were significantly powered by decreased rates of infections (ALT = 1.1% vs 4.4% MS, p=-0.0065). Pneumonia predominates as the main cause of infection following cardiac surgery. It has a reported incidence of up to 8.8% and is also associated with longer ICU admission and subsequent hospital stay [8]. Furthermore, studies have found an independent predictor of pneumonia to be the use of blood transfusions [9,10]. The mechanism by which this occurs is not fully understood; however, it’s suggested that transient periods of immune dysregulation occur following transfusion, thus increasing the susceptibility to infection [10]. Although not assessed in our series, reduced blood loss and therefore the need for transfusion has been formally recognised as a benefit of minimal access cardiac surgery. In their series comparing valvular surgery via right mini-anterolateral thoracotomy versus median sternotomy, Wang et al. found the intra-operative blood loss, transfusion volume, and postoperative chest tube drainage to be significantly reduced (p=0.014, p=0.039, p<0.001 respectively) [11]. Studies analysing minimally invasive direct coronary artery bypass (MIDCAB) versus median sternotomy approaches, including off-pump coronary artery bypass (OPCAB), have replicated these results [12]. The reduced blood loss evidenced in ALT facilitated cardiac surgery is likely due to avoidance of median sternotomy with consequent vascular preservation of the sternal branches of the internal thoracic arteries. Intra-operative and postoperative blood loss have also been found to be predictors of surgical site infection and local hypoperfusion has been implicated as the mechanism behind this [13].

Postoperative pain

Our study found a statistically significant reduction in the number of days that patients took analgesia for in the MICS group (9.7 vs 16.4 days, p=0.049). This result is unsurprising; the smaller incision, reduced tissue traction, and concurrent preservation of bony separation all contribute to the reduced duration of pain. In our centre, MICS is associated with a shortened period of analgesia usage, however, we were unable to quantify the level of pain experienced in relation to the incision due to the retrospective nature of the study. Diegeler et al. found that postoperative pain was higher in their MIDCAB group until postoperative day four, after which patients who underwent CABG via conventional sternotomy reported more pain [14]. The consequences of postoperative pain may be a particularly dangerous sequelae of pulmonary dysfunction. Muscular guarding in response to postoperative pain can manifest with shortness of breath leading to a reduction in tidal volume, vital capacity and consequently a reduction in pulmonary compliance. Furthermore, the negative effects of general anaesthetic on pulmonary mechanics, cough function and airway secretions compound the potential pulmonary effects produced by postoperative pain and the increased blood loss discussed above [15]. Moreover, patients may be prescribed opioids for pain relief and their brainstem-mediated effect may lead to reduced respiratory ventilation which can contribute to the development of pneumonia. Pneumonia is associated with an increased risk of hospital-related mortality (28% vs 6.2%, p=0.003) and diminished long-term survival (62% vs 81%, p<0.0001) [16]. Considering the previous reasons, and the fact that up to 30% of pneumonia cases following cardiac surgery occur in the post-discharge period, the mean reduction of 6.7 days observed in the MICS group of our series is clinically significant and may help to alleviate pneumonia-related prolonged hospital stay or mortality [16]. It must be noted that atelectasis may be caused by single-lung ventilation in surgery with an anterolateral thoracotomy approach, and the insufflation of carbon dioxide into the thorax may worsen this [17]. However, the advantages of single lung ventilatory arrest may include reduced postoperative ventilatory times and earlier mobilisation [18]. It’s plausible that some of the pulmonary effects of single lung ventilatory arrest may be offset by these advantages.

Return to daily activities

Our series found that the period taken to return to daily activities was reduced by 53% in the MICS arm of the study (48.7 days vs 103.2 days, p=0.023). This not only has advantageous consequences for pulmonary rehabilitation, as discussed above, but also in the prevention of thromboembolic events. Furthermore, intensive research into pre-operative depression in cardiac surgery patients has approximated that between 30-40% of patients suffer from some level of dysthymia, a result that is considerably higher than that of the general public [19]. Whilst it’s likely that the symptoms of an individual’s cardiac pathology may contribute to their depression, surgical treatment is not always curative and even with formal psychiatric treatment, the opportunity for cardiac surgery to holistically help a patient should not be ignored. Horne et al. found in their study that postoperative physical inactivity and increased length of stay were independent predictors of depression [20]. The decreased return to daily activities observed in the MICS cohort of our series may carry positive psychosocial implications. Furthermore, three patients (7.5%) in the median sternotomy group in our study reported that they still hadn’t returned to normal daily activities. By achieving normality of daily routine and independence, the individual’s self-esteem, confidence and capacity to perform depression protective activities may be improved. 

Scar satisfaction

Our series found a mean increase of one point associated with scar satisfaction in the MICS cohort compared with the MS group (9.2/10 vs 8.2/10, p=0.032), showing that minimally invasive cardiac surgery tended to improve patient satisfaction with regard to the scar. The question regarding the position of the scar also reached statistical significance. Six patients (15%) in the median sternotomy cohort were bothered about the location of the scar, as compared to zero in the MICS group (p<0.001).

Cosmetically, MICS is perceived to be advantageous with a smaller incision(s) when compared to the median sternotomy. Interestingly, on univariate analysis our study found that in the median sternotomy group, females were less likely to be as satisfied with the scar (females mean score=7.46 vs males mean score=8.56, p=0.031) however, this was not the case when genders were analysed in the MICS group alone. This could be for several reasons. Firstly, larger mammary tissue in women may put strain on the wound, making it more painful, and lead to wound complications such as sternal instability, dehiscence and infection. In fact, after controlling for body mass index, internal mammary artery grafting, diabetes and age, Copeland et al. found an increased odds ratio (OR) for mediastinitis with larger bra sizes (D or DD), when compared with smaller ones (A and B) (OR= 42.1) on multivariate analysis [21,22]. Mediastinitis is a rare but life-threatening complication of median sternotomy which is reported to increase in-hospital mortality by up to four times and may further worsen cosmesis. For obvious reasons, MICS eliminates the risks of sternotomy-associated complications such as subxiphoid incisional herniation and mediastinitis [23]. Secondly, it’s also plausible that more hair on a male chest obscures the scar from view, whereas women are unlikely to have this advantage. Finally, associations with body image may be stronger in females and they may be more conscious of how they look and how others perceive their bodies for various sociocultural reasons. 

Postoperative outcomes, including increased length of stay, increased length of pain period and slower return to daily activities, can be perceived to be stressors on an individual, thus potentially contributing to delayed wound healing. This may be evermore important in the demographics observed in cardiac surgery. Diabetes, obesity and smoking are common pre-operative risk factors for surgical site infection observed in those undergoing an operation on the heart [23]. As found by our study, MICS can improve all the aforementioned markers of postoperative convalescence in a select patient cohort, thus symbiotically decreasing the chances of wound complications. 

Finally, the patients in the median sternotomy group were asked if they were offered their operation via less invasive incisions, whether they would have chosen this over their primary cardiac procedure. The majority (62.5%) said they would. The 27.5% who didn’t commonly said that they were happy with the standard of operation performed already. For some, the primary concern was that they were healthy, symptomless or even just alive and didn’t care about the scar. Naturally, a subset of patients will always exist that have this mindset and this illustrates the importance of always offering both types of operation after information is provided regarding the risks and benefits of both. 

Limitations 

We acknowledge there are several limitations to our study. These include the small number of participants enrolled, and the retrospective nature of the study. The small sample size and single-centre design reduce the study's generalisability. The follow-up period was limited to 12 months. However, we found it was rare for postoperative outpatient follow-up to continue past this point. Randomisation is difficult due to ethical concerns that a patient may not get suitable care, however, data was extracted by an independent data analyst to reduce the risk of bias. It must be stated that the two arms were not propensity matched; therefore, it’s likely that the median sternotomy group may have more clinically unwell patients.

## Conclusions

Minimally invasive cardiac surgery is a rapidly advancing field and has previously been shown to have advantages in a wide range of outcomes including hospital stay, intensive care burden and use of analgesics. Our paper confirms these advantages but also found a statistically significant greater satisfaction level with the scar in the group with MICS incisions. It’s clear MICS is not suitable for everyone, but careful cohort selection can lead to better outcomes for those who undergo this type of surgery. Our preliminary results provide a good basis for further research and warrant confirmation in larger, multi-centre studies. Research is also needed looking specifically at scar satisfaction in cardiac surgery patients and may focus on prospectively evaluating outcomes in this group of patients.

## Appendices

Appendix 1: Questionnaire

**Table 5:** Questionnaire used to assess patient satisfaction.

	Question
1	For how many days after the operation were you taking painkillers? (give answer in days).
2	How soon after the operation could you return to daily activities, unaided? (give answer in days or weeks).
3	How satisfied are you with the aesthetic outcome of the scars from your operation? Rate this from 0 to 10, where 0 is very dissatisfied and 10 is very satisfied.
4	Does the position of the scar bother you? (Please answer yes or no).
Those that underwent midline sternotomy incision were then asked:	
5	If an alternative method were used whereby a smaller incision is made on the side of the chest instead of in the middle, would you have rather undergone this operation? (please answer yes or no).
